# How Do the Multi-Temporal Centroid Trajectories of Urban Heat Island Correspond to Impervious Surface Changes: A Case Study in Wuhan, China

**DOI:** 10.3390/ijerph16203865

**Published:** 2019-10-12

**Authors:** Chen Yang, Qingming Zhan, Sihang Gao, Huimin Liu

**Affiliations:** 1 School of Urban Design, Wuhan University, Wuhan 430072, China; yangchenwhu@whu.edu.cn (C.Y.); sihanggao@whu.edu.cn (S.G.); 2 Collaborative Innovation Centre of Geospatial Technology, 129 Luoyu Road, Wuhan 430079, China; 3 Institute of Space and Earth Information Science, The Chinese University of Hong Kong, Shatin, NT, Hong Kong, China

**Keywords:** thermal environment, spatiotemporal dynamics, impervious surface, trajectories analysis

## Abstract

Conspicuous expansion and intensification of impervious surfaces accompanied by rapid urbanization are widely recognized to have exerted evident impacts on the urban thermal environment. Investigating the spatially and temporally varying relationships between Land Surface Temperature (LST) and impervious surfaces (IS) at multiple scales is of great significance for steering IS expansion and intensification. This study proposes an analytical framework to investigate the spatiotemporal variations of LST and its responses to IS in Wuhan, China at both city scale and sub-region scale. The summer LST patterns in 2002–2017 are extracted by Multi-Task Gaussian Process (MTGP) model from raw 8-day synthesized MODerate-resolution Imaging Spectroradiometer (MODIS) LST data. At the city scale, the weighted center of LST (LSTWC) and impervious surface fraction (ISFWC), multi-temporal trajectories and coupling indicators are utilized to comprehensively examine the spatial and temporal dynamics of LST and IS within Wuhan. At the sub-region scale, urban heat island ratio index (URI), impervious surfaces contribution index (ISCI) and sprawl rate are introduced for further quantifying the relationships of LST and IS. The results reveal that IS and hot thermal landscapes expanded by 407.43 km^2^ and 255.82 km^2^ in Wuhan in 2002–2017 at city scale. The trajectories of LSTWCs and ISFWCs are visually coherent and both heading to southeast direction in general. At the sub-region scale, the specific cardinal directions with the highest ISCI variations are examined to be the exact directions of ISFWC trajectories in 2002–2017. The results reveal that the spatiotemporal variations of LST and IS are highly correlated at both city and sub-region scales within Wuhan, thus testifying the significance of steering IS expansion and renewal for controlling urban thermal environment deterioration.

## 1. Introduction

Unparalleled urbanization in China has led to the more obvious differences of temperature in urban relative to non-urban surroundings, a phenomenon known as the urban heat island (UHI) effect [1,2,3]. The increasing expansion and intensification of impervious landscapes are considered to be non-negligible external forces that intensify the UHI [2,4]. Anabatic urban warming poses threats to environmental sustainability and public health [5,6,7,8]. In this study, UHI is identified as surface urban heat island (SUHIs1) for the emphasis on land surface temperature (LST) [5,6]. The studies of SUHI and its environmental (public health [9], local climate change [2,10], plant phenology [11] and air pollution [12]) and socioeconomic impacts are well-documented [6,9]. Thermal remote sensing has been a powerful tool used in the exploration of LST and SUHI for its broad spatial coverage, frequent revisit cycle and multiple data source [6,13,14]. Furthermore, thermal remotely sensed imageries can vigorously support the SUHI and LST studies for its advantages over in-situ observations as avoidance of non-climatic artifacts (e.g., asynchronous observation time, lack of spatial resolution, non-standing siting) [1,13].

Optimizing the expansion and renewal of impervious surfaces (IS) within urban areas, considering their spatially and temporally varying relationships with LST, is a practical approach to prevent or alleviate urban thermal environment deterioration. As one of the representations of urbanization, the expansion and intensification of IS [15,16] are considered to have significantly modified the radiation fluxes and evapotranspiration within urban areas, thus amplifying SUHI by capturing heat and lessening evaporative cooling [17,18]. There is a commonly recognized fact that LST hotspots are mainly located at impervious landscapes and bare surfaces within cities [19]. Noting the fact that LST and IS are highly correlated, the relationships between LST and IS are well studied in the past several years [15,19,20,21,22]. A positive exponential relationship between IS and LST has been investigated by Xu et al. [22] in Xiamen (China), a subtropical city. Besides, the contribution of IS towards LST variations is claimed to be six times greater than the synergetic contribution of water and green spaces [22,23]. The expansion and intensification of IS within urban areas are generally accompanied by the encroachment of green spaces, thus the deterioration of urban thermal environment and the underlying environmental risks are further exacerbated [24,25]. Investigation of the relationships between LST and IS patterns is essential to facilitate urban landscape planning and management. Notably, the correlation of IS patterns with LST is incompetent to replace the correspondence between LST and other factors, such as urban landscape diversity and composition [24], urban 3-D expansion [9,26] and urban redevelopment [27], etc.

The power of numerous previous studies to provide evidence for urban heterogeneous landscape management is limited because they mainly share two features: (1) the spatial dynamics of LST and IS relationships are emphasized, while the temporal variations of LST are neglected by normally adopting just one snapshot to depict LST patterns in a static state, when in fact, numerous studies have shown that LST patterns are complex and possess significant temporal variability, mainly characterized as diurnal [28], seasonal [10,29], annual [30,31] and inter-annual variations [10]. Besides, the associations between LST and surface factors (including IS) have been verified to be seasonally varying by Liu et al. [29]. The conclusions drawn from a static perspective of LST, a typical geographical and climatic process, can be misleading [20,32,33]. Thus, more considerations have been made to generate typical LST patterns for a specific period, such as average monthly LST [14,34], BLEnding Spatiotemporal Temperatures (BLEST) [35], temporal upscaling [36] and non-parametic models [32,37]. (2) The explorations of the spatiotemporal correlations of LST and IS generally tend to be implemented at a city scale. Zhao et al. [38] have investigated the urban expansion (exactly, IS expansion) in eight cardinal directions from 1984 to 2014 in Shanghai, while its impacts on LST variations are discussed at a city scale. Qiao et al. [39] have depicted the trajectories of center of SUHI and IS in Beijing at a sub-regional scale using distances and angles, but the coupling relationships of SUHI and IS have not been quantified at either the city scale or sub-regional scale. Weng et al. [40] have introduced statistical indicators to quantify the spatiotemporal dynamics of SUHI in eight sub-regions, while the corresponding analysis of surface factors have not implemented. 

Keeping in mind the importance of providing implications for steering urban expansion and intensification from the standpoint of LST and IS correlations at multiple scales either spatially or temporally, this study (1) generates typical summertime-scale LST patterns considering temporal variations of LST using non-parametric Multi-Task Gaussian Process (MTGP) model [32]; (2) quantifies the relationships between LST and IS by integrating moving trajectories and multiple indicators (e.g., spatial coupling indicators [41] and impervious surfaces contribution index (ISCI) [42]) at both the city scale and sub-region scale. The typical summer time LST maps maintain the global diversity and local variation of LST patterns instead of investigating LST and UHI in a static perspective [32,33]. The correlations between IS and LST can provide implications for steering IS expansion and intensification considering the underlying impacts on urban thermal deterioration. Such correlations are believed to facilitate the application of environmental research findings in urban planning and management [24,32,43].

This study proposes an analytical framework to investigate the multi-temporal trajectories of LST and IS as well as their correlations with a geographical focus on the megacity Wuhan, China. Twenty-four MODerate-resolution Imaging Spectroradiometer (MODIS) 8-day synthesized LST subsets from 2002 to 2017 with a 3-year interval are selected. Besides, IS maps with abundant spatial details extracted from a 40-year dataset [44] are adopted. The MTGP model are utilized to generate typical LST patterns in summer days [32]. The analyses in this study has been divided into two parts: (1) at the city scale, characterizing the spatiotemporal variations of LST and IS patterns using weighted center [16,39], moving trajectories [16,39] and spatial coupling indicators [41]; (2) at the sub-region scale, quantifying the coupling relationships of LST and IS in eight cardinal directions using ISCI [42], urban heat island ratio index (URI) [40] and sprawl rate [34,42].

## 2. Study Area and Datasets

### 2.1. Wuhan, China

**Figure 1:**
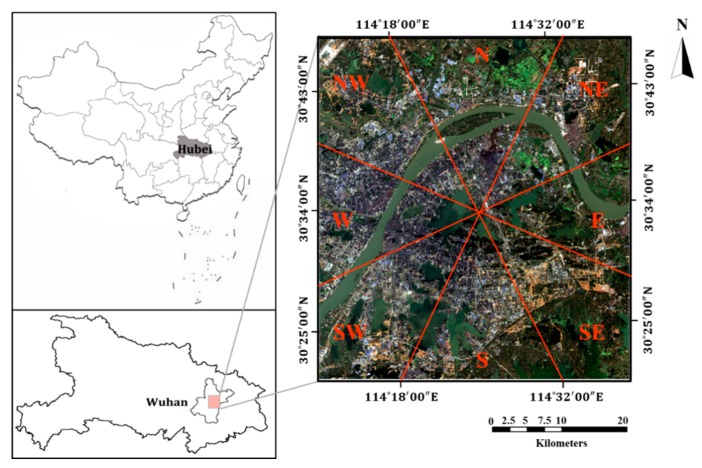
The geographical location and eight sub-regions of the study area and corresponding RGB Landsat-8 OLI image (24 October 2017).

This study investigates the spatiotemporal dynamics of LST and IS as well as their correlations with a geographical focus on Wuhan, China, a megacity located in the middle and lower reaches of the Yangtze River. Wuhan is considered as the “furnace” city in China, with a typical subtropical monsoon climate. In Wuhan, more than a third of the year (specifically, 135 days on average) presents as summer days [10,29]. An unprecedented rapid urbanization (the built-up area within Wuhan has increased from 259.27 km^2^ to 755.09 km^2^ in 1995–2015) in the past two decades has been witnessed in Wuhan, resulting in the significant deterioration of the local thermal environment [33,42]. The spatial extent of the study area is 49 km×44 km. The upper-left and lower-right coordinates are 30°47′32″ N, 114°12′01″ E and 30°20′19″ N, 114°38′32″ E. This study area covers almost all the downtown and suburban districts and is thus appropriate to represent the land composition and transformation of the city. The study area has been divided into eight sub-regions in eight cardinal directions (Figure 1).

### 2.2. Land Surface Temperature (LST) Products

MODIS/Aqua (MYD11A2) V5 LST/E 8-Day L3 Global 1 km Grid products acquired at 13:30 are used to represent the typical LST patterns of summer days in Wuhan in the selected years. The MODIS LST product is a common-used data source in LST and UHI studies [6,45]. The product is generated using the split-window algorithm with an ideal accuracy claimed to be better than 1K [46,47]. The adopted 8-day synthesized LST product is produced by simple averaging, which avoids abundant noise from cloud contamination, snow coverage and other factors [33,34]. Since July and August are investigated to be the hottest months in Wuhan, all 6×4 MODIS 8-day LST subsets (six years in this study, and four subsets for July and August in each year) are utilized to extract the typical summertime LST patterns in this study. Specifically, one raw MYD11A2 subset is selected as the dominant data and three temporally adjacent subsets with the least null observations are adopted as the auxiliary data in each year. The selected LST datasets have been adopted in our previous study [33]. The specific dates of the 24 images in this study are shown in Table 1. The multivariate weather information collected online (https://www.wunderground.com/) of six dominant LST products are listed in Table 2. To ensure the adopted LST products are all desirable, the stability of weather conditions during 8 days of each LST product are investigated in this study [32]. Atmospheric stability plays an essential role in pollutants dispersion [48] and temperature variability [49,50]. Pasquill [51] proposed an easy-to-use method to evaluate the atmospheric stability, taking into account both mechanical turbulence and buoyancy turbulence. This method is of great significance for the investigation towards the relationship between atmospheric dispersion coefficient and categorized stability of boundary layer turbulence [49,51]. The adapted Pasquill-Gifford scale adopted in this study utilizes the in-situ observations of wind speed, cloud cover and sunshine duration to classify the atmospheric stability with multiple parameters [48,49]. The adapted Pasquill-Gifford scale is introduced to ensure the stability of climatic and hydrological conditions during the LST acquirement days. The Pasquill-Gifford scale evaluate the stability of weather conditions in terms of average wind speed and cloud coverage by categorizing the stability into five classes as D (Neutral), E (Slightly Stable), F (Moderately Stable), and G (Extremely Stable) [32]. The utilization of Pasquill-Gifford scale enables us to filter out undesirable LST products considering local atmospheric events and images quality [32,49].

### 2.3. Impervious Surface (IS) Maps

The IS maps are derived from the open-source datasets (http://data.ess.tsinghua.edu.cn/) provided by Gong et al. [44]. This dataset is generated at 30-meter resolution from Landsat satellite imageries with the aid of night-time light (NTL) data on Google Earth Engine (GEE) using the “exclusion/inclusion” algorithm [52] and temporal consistency check algorithm [53]. Firstly, normalized difference vegetation index (NDVI) maps, modified normalized water index (MNDWI) maps and short-wave infrared (SWIR) band are derived from Landsat images using the “exclusion/inclusion” algorithm [52]. The impervious surface thresholds of each year are determined separately. NTL data is then adopted to facilitate the determination the spatial constraints of impervious surfaces. Furthermore, the initial classification results are verified and corrected using temporal consistency check algorithm [53] to avoid unexpected errors caused by temporal non-stationarity. The overall accuracy of IS extractions is claimed to be higher than 93% [44]. More detailed information can be checked in the reference [44]. In this study, the IS maps at 30-meter resolution in 2002, 2005, 2008, 2011, 2014 and 2017 are extracted from the dataset. Furthermore, the impervious surface fraction (ISF) data has been calculated using 500 m×500 m gridded fishnet on the ArcGIS 10.2 software platform (Environmental Systems Research Institute Inc., Redlands, CA, USA) for the ISF weighted center identification [16].

### 2.4. Methodology

**Figure 2:**
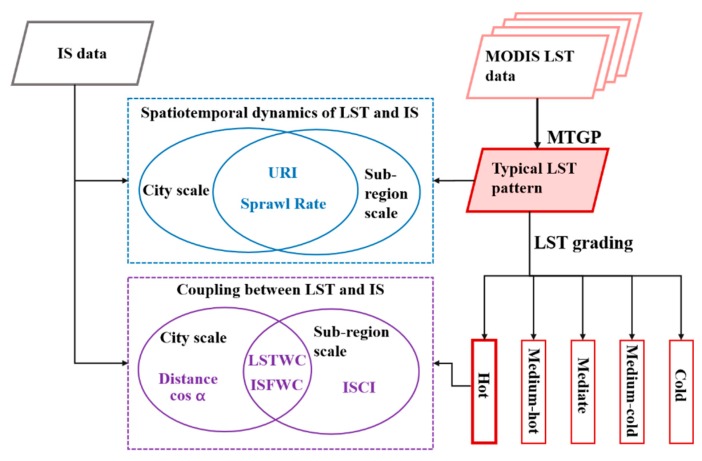
The technical flow of this study.

The analytical framework proposed in this study can be summarized as a technical flow represented in Figure 2. It includes four principle steps: (1) generate typical LST patterns with noise removed and missing observations filled in summer using MTGP (Section 3.1); (2) categorize the thermal landscapes into five classes as hot, medium-hot, mediate, medium-cold, cold by LST grading (Section 3.2); (3) investigate the spatiotemporal dynamics of LST and IS at both the city scale and sub-region scale, using the weighed center of IS (ISFWC in Section 3.3) and LST (LSTWC in Section 3.4) as well as sprawl rate (Section 3.5) of IS expansion and hot thermal landscape variations; (4) quantify the coupling relationship between LST and IS using coupling indicators (Section 3.6) at the city scale and impervious surfaces contribution index (ISCI in Section 3.7) at the sub-region scale.

#### 2.4.1. Multi-Task Gaussian Process (MTGP) Model for Typical LST Patterns Extraction

In this study, the non-parametric Multi-Take Gaussian Process (MTGP) model is used to extract typical LST patterns in summer using one raw MODIS LST map with the auxiliary information in three temporally adjacent LST maps [32,33]. It is generally acknowledged that raw remotely-sensed LST products suffers from noises and null pixels resulted by undesirable weather conditions, atmospheric interferences or observation failures [54,55]. It can be assumed that the typical LST distribution of a specific site is a potential pattern hidden in the raw LST products suffered from discrete noises, which needs to be recovered [37,55,56]. The benefits of introducing MTGP into this study are three-folds: (1) the missing observations are filled and the poor-quality pixels around cloud coverage and noises are smoothed [10,55], (2) the noise-free and continuous LST patterns support pattern analysis more effectively [57], and (3) the extracted summertime LST patterns by MTGP can capture the typical patterns for sharing information across multiple images, instead of considering LST in a static view [32,33,36]. In this study, the produced typical LST patterns are interpolated into 500-meter resolution, which approximates to the optimal scale towards LST studies at urban scale [20,58]. Besides, the interpolated LSTs at finer resolution containing more spatial details are believed to support pattern recognition better and can benefit the local LST anomalies characterization [20,33].

The observed LST dataset can be defined as $D=\left\{\left(x_{i},t_{ij}\right)|i=1,\ldots ,n,j=1,\ldots m\right\}$, *n* is the number of pixels on one image, and *m* is the number of images applied in the model. The Gaussian process (GP) model generalizes the extraction form to a vector [$f_{1}$,…, $f_{n}$]*^T^* of infinite length, where the vector of any finite set is joint Gaussian. Model $f\left(x\right)\sim GP\left(m\left(x\right),K^{f}K^{x}\right)$ is completely defined by mean function m(x) and covariance function. Specifically, $K^{f}$ represents the covariance function of inter-task information between images, and $K^{x}$ represents the covariance function of inter-task information within images.

The typical LST pattern $\bar{f^{*}}$ can be predicted as:

(1) $$\bar{f^{*}}=m\left(x^{*}\right)+{\left(k^{f}⊗k^{x}\left(x^{*},x\right)\right)}^{T}{\left(K^{f}⊗K^{x}+\Delta ⊗I\right)}^{-1}\left(t-m\left(X\right)\right),$$

where $f^{*}$ is the latent LST value predicted by MTGP, $x^{*}$ represents the test input, ⊗ is the Kronecker product of matrices, and Δ is a diagonal matrix recording noise $\sigma^{2}$.

#### 2.4.2. LST Grading

The thermal landscapes in both urban areas and suburban surroundings have been classified into five categories as Cold, Medium-cold, Median, Medium-hot and Hot using the mean-standard deviation (STD) method (Table 3) [14]. The hot thermal landscape extracted using LST grading has been testified to highly correlated to IS coverage [14,19]. Thus, in the following sections, the hot thermal landscape shall perform as basic role for LST spatiotemporal dynamics investigation (Section 4.2) and LST-IS coupling relationships quantification (Section 4.3).

### 2.5. Weighted Center of LST and IS

#### 2.5.1. Impervious Surface Fraction Weighted Center (ISFWC)

The impervious surface fraction weighted center (ISFWC) is introduced by Xu et al. [16] to reveal the urban expansion orientation with impervious surface fraction (ISF) maps. The ISFWC is calculated as:

(2) $$\{\begin{array}{c} \bar{x}=\frac{\sum_{i=1}^{n}f_{i}x_{i}}{\sum_{i=1}^{n}f_{i}}\\ \bar{y}=\frac{\sum_{i=1}^{n}f_{i}y_{i}}{\sum_{i=1}^{n}f_{i}} \end{array},$$

where $\bar{x},\bar{y}$ are ISFWC coordinates, i represent the i-th pixel, n is the total amount of pixels in an ISF map, $f_{i}$ is the ISF of i-th pixel. In this study, the ISF maps from 2002 to 2017 are calculated using 500 m×500 m gridded fishnet on ArcGIS 10.2 platform based on corresponding IS images at 30-meter resolution provided by Gong et al. [44].

#### 2.5.2. LST Weighted Center (LSTWC)

The spatiotemporal dynamics (moving directions and trajectories) of LST are depicted using LST weighted center (LSTWC), the modified ISFWC considering the LST patterns. The coordinates of LSTWC are calculated as:

(3) $$\{\begin{array}{c} \bar{x}=\frac{\sum_{i=1}^{n}m_{i}x_{i}}{\sum_{i=1}^{n}m_{i}}\\ \bar{y}=\frac{\sum_{i=1}^{n}m_{i}y_{i}}{\sum_{i=1}^{n}m_{i}} \end{array},$$

where $\bar{x},\bar{y}$ are the coordinates of calculated LSTWC x,y are the coordinates of a specific pixel, and $m_{i}$ is the difference between LST value of ith pixel and mean LST values of the city. The moving trajectories of LSTWC in 2002–2017 reflect the general trends of LST variations in Wuhan.

### 2.6. Quantification of Spatiotemporal Dynamics and Relationships between LST and IS

#### 2.6.1. Urban Heat Island (UHI) Ratio Index

The urban heat island ratio index (URI) is used to quantitatively characterize the UHI intensity within the study area in this study [22,39,40]. URI index is defined as follows:

(4) $$\mathrm{URI}=\;\frac{1}{100m}\sum_{i=1}^{n}w_{i}p_{i},$$

where m is the number of thermal landscape categories classified using the method in Section 3.2 (m=5 according to Table 3), n is the number of thermal landscapes with higher LST values than the mediate thermal landscapes (n=2 in this study), $w_{i}$ is the weight of a specific thermal landscape (Cold = 1, Medium-cold = 2, Median = 3, Medium-hot = 4 and Hot = 5) and $p_{i}$ is the coverage proportion of the corresponding thermal landscape. The range of URI is from 0 to 1, and a higher URI index indicates that the UHI effect is more severe.

#### 2.6.2. Sprawl Rate

In this study, the sprawl rates of hot thermal landscape (identified in Section 3.2) and IS are quantified. This sprawl rate can be calculated to reveal the expansion intensity of hot thermal landscape and IS in specific regions and periods. The sprawl rate is defined as:

(5) $$V_{t}=\frac{S_{t}}{S_{t-1}},$$

where t denotes the specific year, t−1 is the previous time of t. For example, when t equals to 2005, t−1 shall be 2002. $S_{t}$ and $S_{t-1}$ are areas of hot thermal landscape or IS in t or t−1, respectively.

#### 2.6.3. Coupling Indicators between IS and LST

To quantitatively evaluate the coupling relationships between ISFWCs and LSTWCs in the spatial extent, the coupling indicators are utilized in this study [41]. The coupling indicators are capable to measure the spatial distance of ISFWCs and LSTWCs and the azimuths of the moving trajectories of ISFWCs and LSTWCs, respectively. The closer spatial distance of LSTWCs and ISFWCs and smaller angle between trajectories of LSTWCs and ISFWCs reveal the stronger coupling relationship between LST and IS in the study area. The spatial distance and azimuth are calculated as:

(6) $$D=\sqrt{{\left(X_{ISFWC,t}-X_{LSTWC,t}\right)}^{2}+{\left(Y_{ISFWC,t}-Y_{LSTWC,t}\right)}^{2}},$$

(7) $$\cos\alpha =\frac{\Delta X_{ISFWC}\times \Delta X_{LSTWC}+\Delta Y_{ISFWC}\times \Delta Y_{LSTWC}}{\sqrt{\left(\Delta X_{ISFWC}^{2}+\Delta Y_{ISFWC}^{2}\right)\times \left(\Delta X_{LSTWC}^{2}+\Delta Y_{LSTWC}^{2}\right)}},$$

where D is the spatial distance between LSTWC and ISFWC at a specific time. And α is the angle between moving trajectories of LSTWCs and ISFWCs. $X_{ISFWC,t}$ and $Y_{ISFWC,t}$ are coordinates of ISFWCs at a specific time t ($X_{LSTWC,t}$ and $Y_{LSTWC,t}$ share the similar meaning). $\Delta X_{ISFWC}$ and $\Delta Y_{ISFWC}$ are the coordinates differences of ISFWC between the locations at a specific time t with the previous time t−1, respectively (e.g., $\Delta X_{ISFWC}=X_{ISFWC,t}-X_{ISFWC,t-1}$). $\Delta X_{LSTWC}$ and $\Delta Y_{LSTWC}$ are the coordinates differences of LSTWC. The data range of cosα is (−1,1). The cosα equals to −1 reveals that the angle between trajectories of LSTWCs and ISFWCs at a specific time equals to 180°, and cosα equals to 1 reveals that the angle equals to 0°.

#### 2.6.4. Impervious Surface Contribution Index

The contribution of IS expansion to the LST variation in eight cardinal directions from 2002 to 2017 are quantified using impervious surface contribution index (ISCI) as follows [34,42]:

(8) $$ISCI=\left(LST_{IS}-LST_{mean}\right)\times P_{IS},$$

where ISCI is the impervious surface heating contribution in a specific region, $LST_{IS}$ is the average LST of impervious surfaces in the region, $LST_{mean}$ is the mean LST value of the city, and $P_{IS}$ is the proportion of impervious surfaces in the region. $P_{IS}$ varies from 0 to 1 in this study.

## 3. Results and Discussion

### 3.1. Impervious Surfacve Expansions within Wuhan

**Figure 3:**
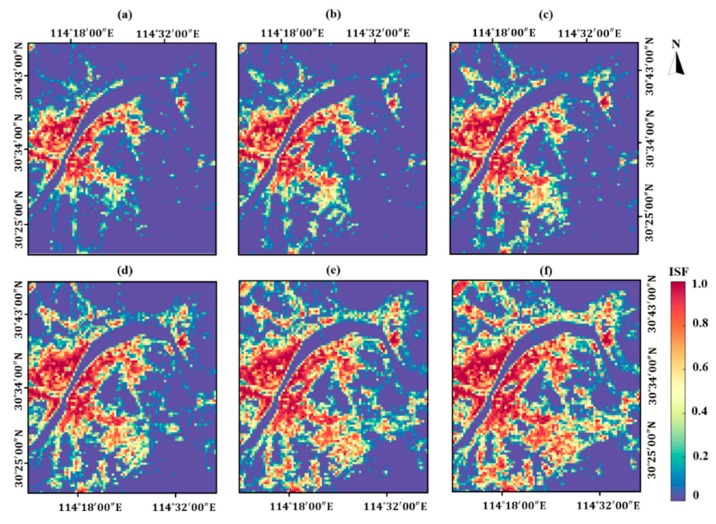
The impervious surface fraction (ISF) maps from 2002 to 2017 in the study area. (**a**) 2002; (**b**) 2005; (**c**) 2008; (**d**) 2011; (**e**) 2014; (**f**) 2017.

The ISF maps in Wuhan from 2002 to 2017, as presented in Figure 3, show an obvious expansion trend from 2002 to 2017. In the period of 2002–2008, the expansion of IS mainly has occurred in the East Lake High-Tech Development Zone located in Hongshan District. From 2008 to 2014, in addition to East Lake High-Tech Development Zone, the expansion of IS can also be seen in the Tianhe Airport in the northwest corner of the study area, Yangluo new town in the northeast corner of the study area and Jiangxia district in the south of the research area. In 2017, the IS coverage has been expanded without directivity into suburban surroundings, such as Huashan new town in the east and southeast of the area, the Tianhe Airport and its surroundings as well as the Yangtze River new town lies in the north of the study area.

**Figure 4:**
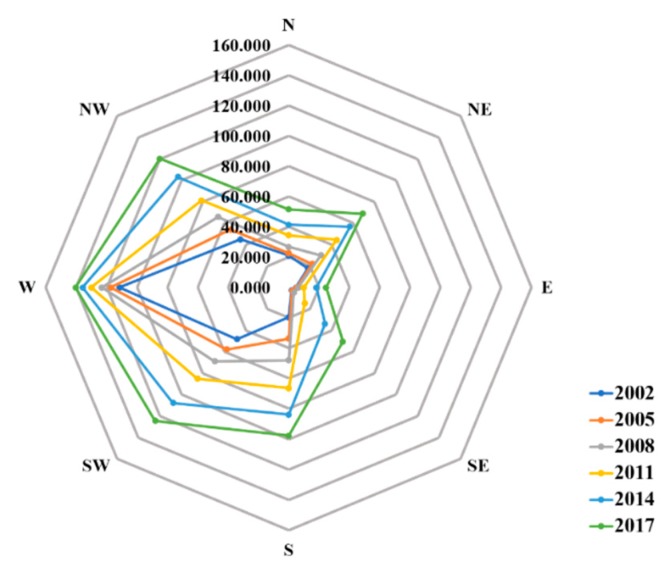
The variations of impervious surface area in eight sub-regions from 2002 to 2017.

The detailed statistics of the IS expansion are reported in Table 4. The IS coverage has significantly expanded from 270.75 to 678.18 km^2^ in the period of 2002–2017. The expansion of IS occurs in a gradually increasing manner from 2002 to 2011 (sprawl rate up to 1.25), while decelerating from 2014 to 2017 (sprawl rate decreased from 1.25 to 1.17). At the sub-regional scale, the IS expansion and the sprawl rates of IS in eight cardinal directions are respectively shown in Figure 4 and Table 5. The west sub-region has the most IS coverage (114.3 km^2^ in 2002 and 140.36 km^2^ in 2017), but the area growth of IS in the west sub-region is the least significant (overall rate equals to 18.22).

The west sub-region mainly includes Jianghan district, Qiaokou district and Hanyang district, which have been the main downtown area of Wuhan since the 1950s [59]. Previous studies have revealed that this area has been mainly updated internally from 2000 to 2015 without significant expansion [59,60]. The southeast region has experienced the most significant IS expansion in the study area. Specifically, the IS coverage of this sub-region is the least among the eight sub-regions in 2002 (2.77 km^2^), while the IS area in southeast sub-region have increased remarkably to 109.99 km^2^ with an overall rate of 18.22 (up to 3.19 in the period of 2008–2011) from 2002 to 2017.

### 3.2. Spatiotemporal Dynamics of LST Patterns

The monthly LST patterns in summer days from 2002 to 2017 are extracted by the heuristic MTGP model using one raw MODIS LST subset with three temporally adjacent auxiliary LST maps. The accuracy of monthly LST patterns has been verified to be within 1 °C (0.5 °C in most cases) in the previous studies [10,32,33]. 

**Figure 5:**
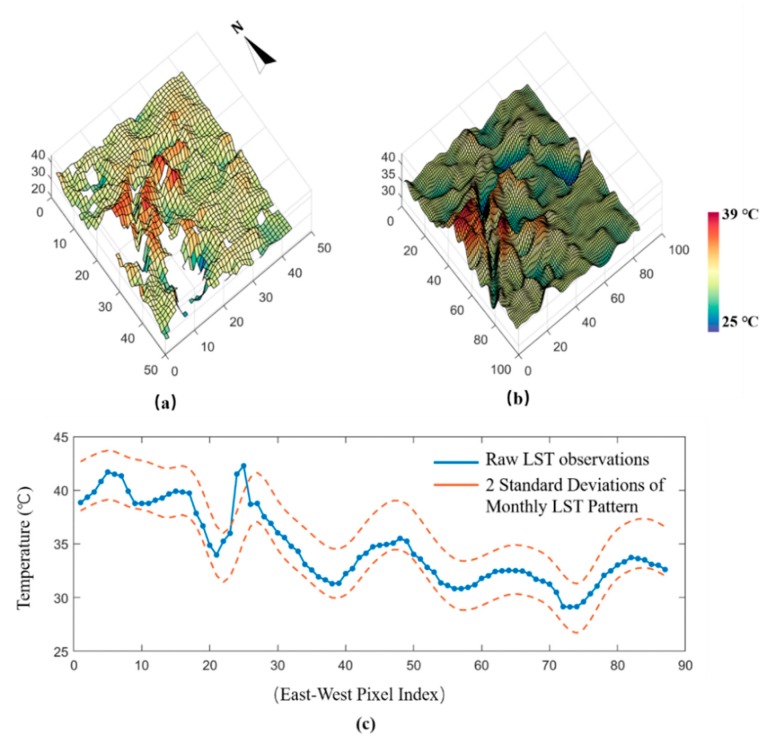
(**a**) The raw MODIS land surface temperature (LST) product of 4 July 2002. (**b**) The corresponding monthly LST pattern extracted by Multi-Task Gaussian Process (MTGP) model. (**c**) Accuracy assessment of extracted monthly LST pattern.

The typical summertime LST pattern extraction and accuracy evaluation in this study is exemplified using raw MODIS LST product on July 4th 2002 as shown in Figure 5. The continuous and noise-free LST map (Figure 5b) with typical summertime LST pattern has been recovered from the cloud contaminated image using MTGP, which is claimed to support local LST anomalies investigation better [20,33]. In such operation, three LST products acquired on July 12th, 2018, August 21th, 2018 and August 28th, 2018 are utilized as auxiliary data in typical summertime LST patterns extraction. The MTGP has been conducted in all six years based on six dominant images (one in a year) with eighteen auxiliary LST products (three in a year). Furthermore, Figure 5c shows that all the raw LST observations are within the two standard deviation (STD) of monthly LST pattern. It reveals that the extracted LST patterns can reflect the typical monthly LST pattern with an acceptable accuracy [20,33].

The accuracy of all six monthly LST patterns are evaluated using STD, bias [61] and correlation coefficient (CC) [33,62]. The detailed assessments of monthly LST patterns are reported in Table 6. As reported in Table 6, the biases of six LST patterns are all within two STD. The maximum and minimum bias values are 0.15 °C and 0.48 °C, and the CC values are all larger than 0.96. The accuracy assessments demonstrate that the monthly LST patterns are generated with ideal accuracy.

**Figure 6:**
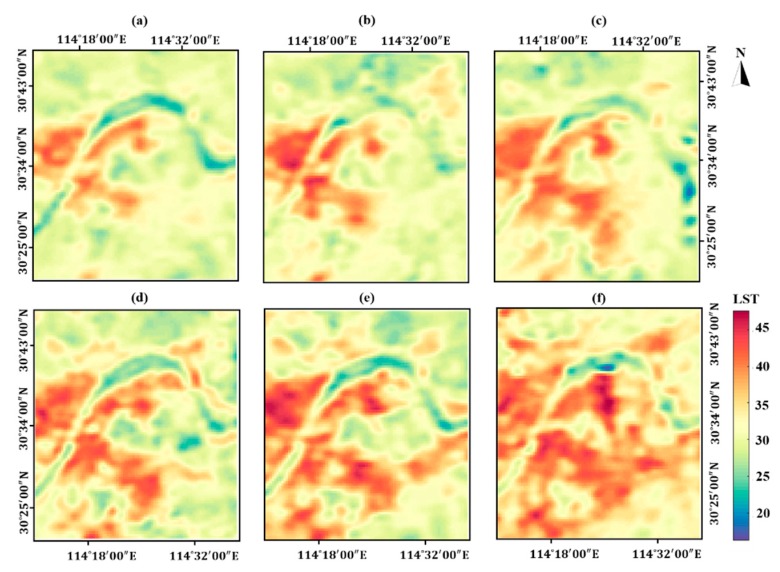
The MTGP extracted monthly LST patterns in Wuhan from 2002 to 2017. (**a**) 2002; (**b**) 2005; (**c**) 2008; (**d**) 2011; (**e**) 2014; (**f**) 2017.

**Figure 7:**
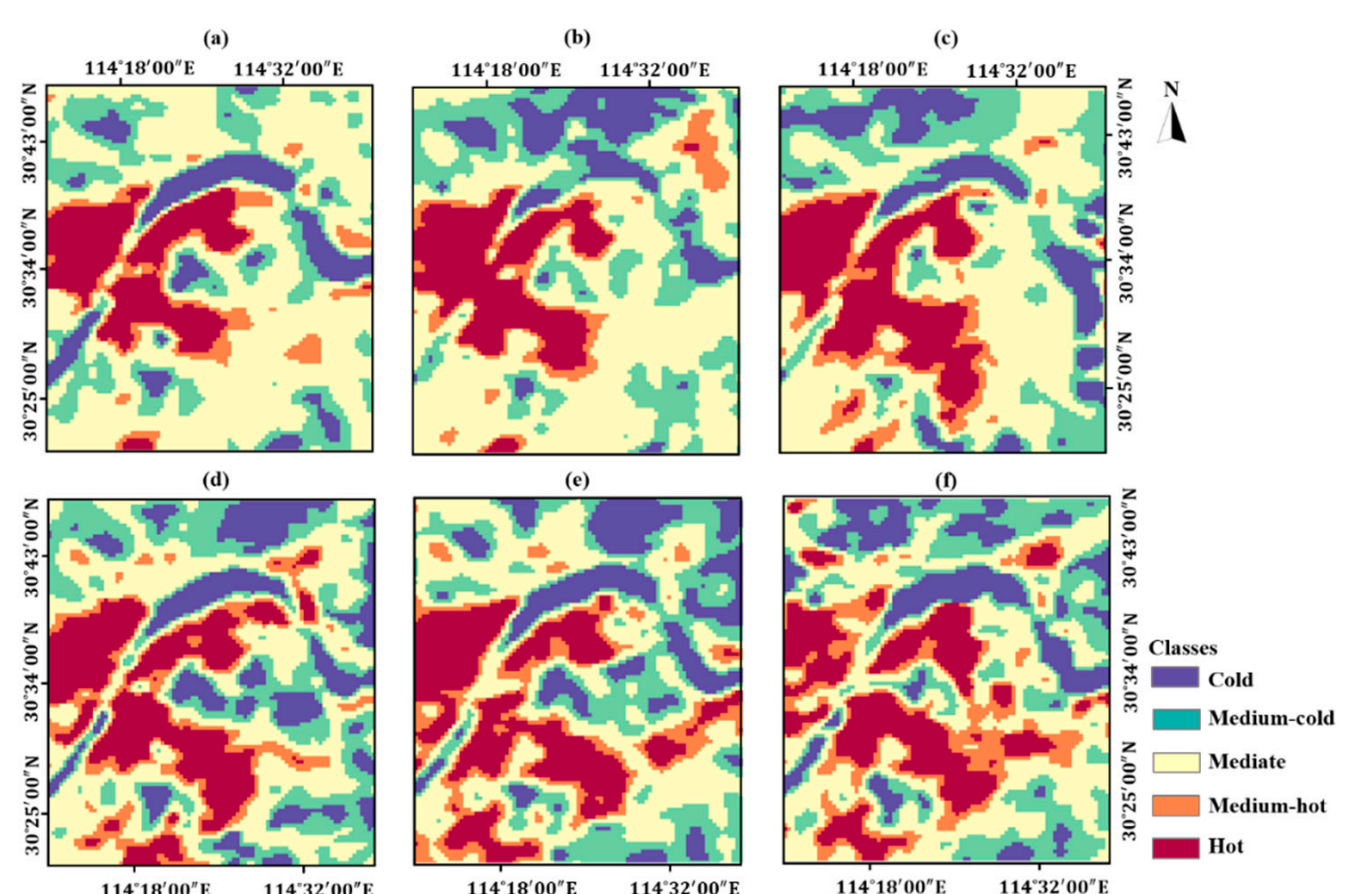
The five categories of thermal landscapes in Wuhan from 2002 to 2017. (**a**) 2002; (**b**) 2005; (**c**) 2008; (**d**) 2011; (**e**) 2014; (**f**) 2017.

The monthly LST patterns and classified thermal landscapes are shown in Figure 6 and Figure 7, respectively. Visually, the distributions of hot thermal landscape in six years are quite consistent with that of IS. The direction of hot thermal landscape expansion within the study area is southeast in general. 

Specifically, the thermal landscape in the Huashan new town and the Tianhe Airport transformed from medium-hot into hot in 2017, about three years after IS expansion witnessed in such areas. Furthermore, hot thermal landscape can be seen in Huashan new town in the east part of the study area. Referring to ISF maps in Figure 3, 2014 is the specific year when ISF in Huashan new town increased significantly.

As reported in Table 7, the areas of hot thermal landscape in the city have expanded significantly from 276.09 to 531.91 km^2^ during 2002–2017. The increased URIs from 2002 to 2017 indicate that the UHI effect of the city has become more extensive [39,40]. Specifically, the URI of east sub-region have increased significantly from 0.0001 in 2014 to 0.14 in 2017. The URIs of west sub-region were all greater than 0.61, indicating the UHI effect of the west sub-region was the most extensive among the eight sub-regions. During 2002–2008, a 0.06 increase of hot thermal landscape sprawl rate has been witnessed in the city. However, the sprawl rate of hot thermal landscape fluctuated in the period of 2011–2017. Besides, URI at the city scale increased one time (from 0.13 to 0.25) in this period.

**Figure 8:**
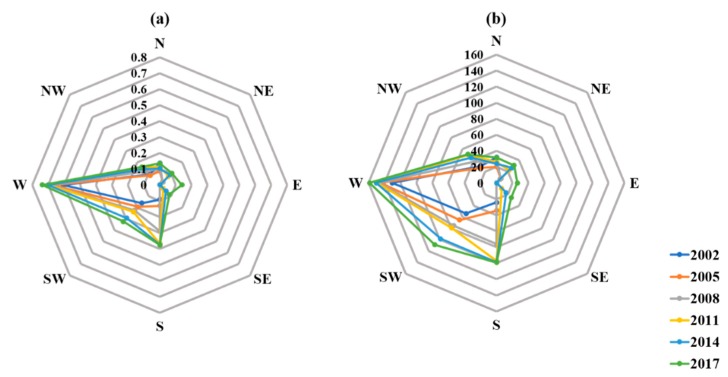
The variations of the urban heat island ratio index (URI) and hot thermal landscape area in eight sub-regions from 2002 to 2017. (**a**) URI; (**b**) Hot thermal landscape.

At the sub-region scale, there was no hot thermal landscape distribution in the east and southeast sub-regions before 2011, thus URIs in the east and southeast sub-regions were equal to zero before 2011 (shown in Figure 8). From 2011 to 2017, the hot thermal landscape area in the southeast sub-region increased from 9.12 to 26.15 km^2^. In addition, in the eastern sub-region, 0.11 km^2^ of hot thermal landscape appeared for the first time in 2014, and then significantly increased to 26.04 km^2^ in 2017.

The sprawl rates of hot thermal landscape in the eight sub-regions are generally greater than 1, which indicates that hot thermal landscape keeping expanded in 2002–2017 (Table 8). The sprawl rates of the northern and northwestern regions were less than 1 from 2002 to 2005 and from 2011 to 2014, indicating that the hot thermal landscape areas of these two regions decreased in above periods. The east sub-region has experienced a flying increase of sprawl rate during 2014–2017, which is consistent with the former discussion. Besides, in the period of 2008–2011, the hot thermal landscape sprawl rate was up to 2.86 in the northeast sub-region. Referring to Figure 7, this variation can be ascribed to the emergence of a hot thermal landscape in the Yangluo new town in 2011.

### 3.3. The Multi-Scale Correlations between LST and IS

**Figure 9:**
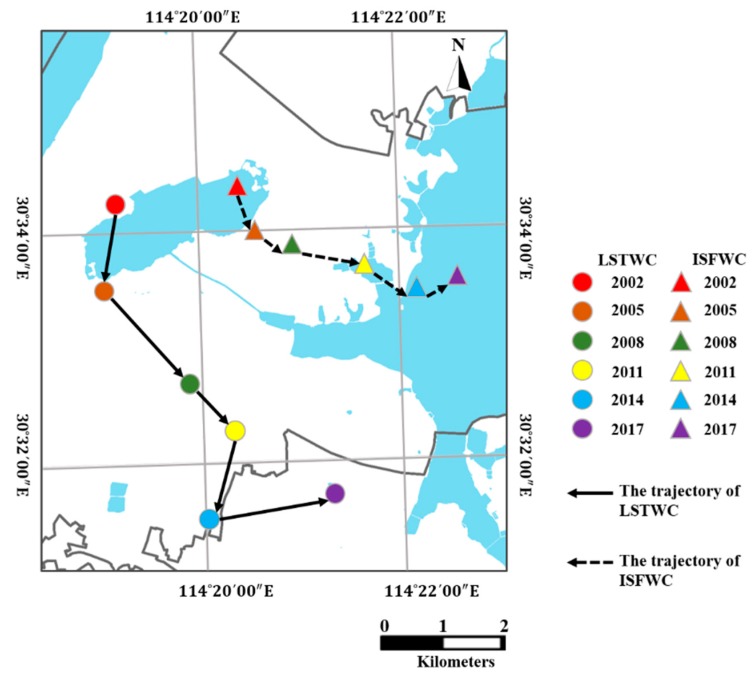
The moving trajectories of LSTWCs and ISFWCs from 2002 to 2017.

The strong correlations between LST and IS/ISF are well documented in previous studies [15,20,22,38,39,40]. In this study, the contributions of IS variations towards LST are quantified at both city scale and sub-region scale using multiple indicators. At the city scale, the weighted gravity center of LST (LSTWC) and impervious surface (ISFWC) are adopted to reveal the spatiotemporal dynamics of LST and IS in this study. The specific locations and moving trajectories are shown in Figure 9.

As shown in Figure 9, the movement trajectories of LSTWCs and ISFWCs are quite similar and the overall trends heads the southeast. But the trajectory of ISFWCs and LSTWCs do not match in 2005 and 2014. Such discords can be ascribed that the warming contributions of IS expansions are geographically and temporally varying, i.e., the same amount of IS expansion at different geographical locations or different times may not contribute the same to the variations of urban thermal environment [40,42]. 

**Figure 10:**
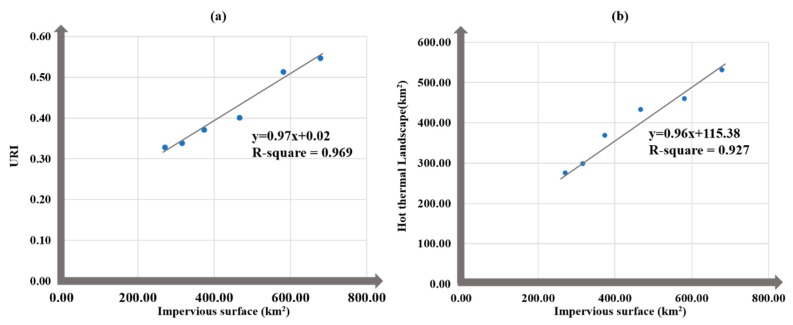
(**a**)The relationship between IS and URI; (**b**) The relationship between IS and hot thermal landscape.

The warming contribution of IS expansion will be discussed in the subsequent paragraphs using ISCI. Specifically, LSTWC moved to the southwest while ISFWC moved to the southeast during 2002–2005 and 2011–2014. At the city scale, the variations of LSTWCs are generally more significant than that of ISFWCs in the perspective of distances and azimuths. Besides, a positive linear relationship (R^2^ = 0.969) between IS and URI has been explored at the city, revealing that the IS expansion has been resulted in the emerge of UHI effect in Wuhan (Figure 10a). Furthermore, the hot thermal landscape is highly correlated to IS (R^2^ = 0.927) in the study area (Figure 10b), indicating that the expansion of hot thermal landscape can be attributed to the expansion of IS.

At the city scale, the coupling relationship of LST variations and IS expansions are further quantified by the distance of LSTWC and ISFWC as well as the cosine of the angle (cos α) between LST trajectories and ISFWC trajectories. As reported in Table 9, the distances between LSTWCs and ISFWCs have been increased from 1944.49 meters to 5047.76 meters in the period 2002–2014, and have been decreased to 3948.09 meters in 2017. Correspondingly, cos α between trajectories of LSTWC and ISFWC have decreased significantly from 0.925 to 0.254 during 2002–2014 and then have increased to 0.709 in 2017. The quantitative results reveal that the coupling relationship between IS expansions and LST dynamics is quite strong (cos α larger than 0.709 and distances between LSTWCs and ISFWCs shorter than 3948.09 meters in general) in the period of 2002–2017. But the coupling relationship have been weakened at the city scale.

The weakened coupling relationship can be partially attributed to the LST decrease in downtown areas within Wuhan explored in the previous study [10] (especially the Qingshan industrial park, which experienced the hottest LST within Wuhan from 2002 to 2017). Furthermore, the landscape composition and diversity are explored to be highly correlated to the LST patterns in the context of urbanization [24,25]. As one of the central cities in China, Wuhan has experienced not only extensive expansion, but also remarkable landscape renewal during the study period [42,63]. The decreased UHI effects within downtown Wuhan, induced by landscape renewal [42], can be partially responsible for the weakened coupling relationship between IS and LST in the study area.

At the sub-region scale, the heating contribution of IS have been quantified using impervious surface contribution index (ISCI) introduced in Section 3.7. As reported in Table 10, all the sub-regions possess ISCIs larger than zero, indicating all the eight sub-regions are experienced the positive warming contribution [42]. 

During 2002–2011, the west sub-region with the most coverages of hot thermal landscape and IS possessed the most significant positive contribution to the UHI effect of the city. However, the ISCI of the west sub-region was not the highest, and the southwest sub-region ranked the first with ISCI value of 112.58. And in 2017, the north sub-region possessed the highest ISCI value of 105.40. Generally, the ISCIs in all sub-regions have experienced significant increase from 2002 to 2017, especially the southeast sub-region with a remarkable increase of 45.29. Such increases indicate that the warming contribution of IS towards UHI effect in Wuhan has become more intensive in the period of 2002–2017. 

The variations of ISCIs in the sub-regions from 2002 to 2017 are listed in Table 11. The variations of ISCIs can be regarded as the potential driving forces of the LSTWCs movements [39,42]. As reported in Table 11, the impervious surfaces warming contribution within west sub-region has dramatically decreased by 68.36 during 2002–2017. The ISCIs increases of the northeast, east and southeast sub-regions are 36.54, 26.95 and 27.74, indicating the IS warming contributions of these sub-regions have been significantly strengthened in this period. Generally, during each period, the LSTWCs have moved in the same direction as ISCIs have the most positive increase. In 2002–2005, the southwest sub-region has a positive ISCI increase of 37.90, and LSTWCs exactly move towards southwest direction. Such consistency can be witnessed in the period of 2005–2008, 2008–2011, 2011–2014 and 2017. Furthermore, the southeast sub-region possesses the most significant increase of ISCI value (an increase of 27.74), which is consistent with the overall southeast directions of LSTWCs movements at the city scale during 2002–2017. This quantitative warming contribution results reveal that the spatiotemporal dynamics of LST landscapes has strong coupling relationships with IS expansions at the sub-region scale. And the variation of impervious surface contribution has been explored to be highly correlated with the movements of LSTWCs.

### 3.4. Implications and Limitations

The deterioration of urban thermal environment poses more serious influences towards public health [7,9,64], e.g., thermal comfort degradation [65,66], mental illness [67], extreme heat-related morbidity and mortality [68], especially for vulnerable city dwellers such as the elderly population, juveniles and children, outdoor workers and low-income groups [9,55,66,69]. A better understanding of thermal center movements and orientations corresponding to IS variations can facilitate the decision-making towards steering urban expansion and intensification and public health improvement [69,70,71]. This study has quantified the coupling relationships between LST and IS at both the city scale and sub-region scale. Such multi-scale quantitative analysis can bridge the gap between local climate studies and urban planning by providing implications for better managing the land use/land cover changes within cities considering their underlying impacts on local thermal environment and public health. Furthermore, the LST and UHI maps can be integrated with urban morphological indicators (e.g., sky view factor) [29,72], local climate zone (LCZ) [9,73], socioeconomic and demographic factors to estimate heat exposure risk [9]. On the basis of existing studies, the continuous exposure to hot temperature could increase health risks and energy consumption [8,55,74]. Therefore, how to regulate the movements and orientations of urban thermal center in response to the distribution of urban construction center, population center and economic center is of great significance for urban public health preparedness oriented to the urban sustainability and resilience improvement [45,75].

However, there are still some uncertainties in this study. The 3-year temporal internal may not be the optimal temporal scale. As scale is claimed to be crucial for all ecological and geographical studies [51], the variations of LST and its responses to IS should be investigated at multi-temporal scales in the further studies. However, this study does not implement multi-temporal scale investigations to identify the optimal temporal scale for the comparative explorations of LST-IS relationships. Furthermore, urban renewal is not only two-dimensional (2-D) expansions such as IS expansion and intensification, but also accompanied by conspicuous three-dimensional (3-D) expansion [26,72] (attached to variations of urban forms) and urban function transformations [9,29]. In this study, only the relationships between LST and satellite imageries derived IS maps are examined. The interactions between LST and other surface factors should be emphasized in future studies, especially factors reflecting urban metabolisms that cannot derived from remotely sensed images [24].

## 4. Conclusions

The multi-temporal trajectories of LST and IS as well as their underlying relationships have been comparatively investigated at both the city scale and the sub-region scale in Wuhan (China). The major findings can be summarized as follows:

(1) The hot thermal landscapes of the study area have significantly expanded from 276.09 to 531.91 km^2^ and the impervious surfaces has expanded by 407.43 km^2^ (from 270.75 to 678.18 km^2^) at the city scale. There is a positive linear relationship between the expansions of hot thermal landscape and IS (R^2^ = 0.969).
(2) URIs have increased from 0.33 to 0.55, indicating the UHI effect of the study area have become more intensive in the period of 2002–2017. The increase of URIs is highly correlated to the expansion of IS at the city scale (R^2^ = 0.927).
(3) The most expansion of hot thermal landscape has been witnessed in the east, southeast and northeast sub-regions, which is quite consistent with the IS expansions at the sub-region scale.
(4) At the city scale, the coupling relationship between LST and IS is quite strong (cos α larger than 0.709 and distances between LSTWCs and ISFWCs shorter than 3948.09 meters in general). However, the coupling relationship has been weakened in 2002-2014, afterwards strengthened in 2017.
(5) At the sub-region scale, the warming contribution of impervious surfaces has been examined to be the external forcing of the movements of LSTWC. Specifically, LSTWC tend to move towards the sub-region with the most significant variation of impervious surfaces contribution index. Implications and suggestions are available for the decision makers to steer land use/land cover and allocate urban sprawl based on the findings of this study.

Although the heuristic MTGP is capable of generating typical summertime LST patterns by integrating four temporally adjacent LST maps, the long-term variations of LST and the optimal temporal scale of time-series LST data deserve consideration in the further studies. Furthermore, IS variations are incompetent to interpret the spatiotemporal dynamics of LST solely. The interactions between LST variations and other surface factors should be further quantified. The impacts of urban form and urban function on LST within cities should be emphasized in the future studies.

## Figures and Tables

**Table 1:** The dates of LST products of each year in this study.

Year	Date of the Dominant LST Product	Dates of the Auxiliary Products
2002	July 4th	July 12th
		August 21th
		August 29th
2005	July 12th	July 20th
		July 28th
		August 5th
2008	August 21th	July 11th
		July 19th
		July 27th
2011	August 13th	July 4th
		July 20th
		August 29th
2014	July 28th	July 20th
		August 5th
		August 13th
2017	July 12th	July 20th
		August 13th
		August 21th

**Table 2:** Weather information of the selected dominant land surface temperature (LST) products.

Selected Date	8-Day Average Air Temperature (°C)	8-Day Average Relative Humidity	Average Wind Force (m/s)	Average Cloud Cover	Adapted Pasquill-Gifford Stability Class
2002/07/04	33.42	68.62	1.19	2.61/8	G
2005/07/12	32.71	65.68	1.64	2.34/8	G
2008/08/20	30.86	81.52	1.94	3.57/8	G
2011/08/13	33.07	67.59	2.56	1.82/8	F
2014/07/28	34.12	74.64	3.89	3.67/8	E
2017/07/12	34.35	67.38	2.30	2.16/8	F

**Table 3:** Thermal landscape classification using mean-standard deviation (STD) method.

Thermal Landscape	LST Range
Hot	$T\left(x,y\right)\geq T_{mean}+STD$
Medium-hot	$T_{mean}+0.5\times STD\leq T\left(x,y\right)<T_{mean}+STD$
Median	$T_{mean}-0.5\times STD\leq T\left(x,y\right)<T_{mean}+0.5\times STD$
Medium-cold	$T_{mean}-STD\leq T\left(x,y\right)<T_{mean}-0.5\times STD$
Cold	$T\left(x,y\right)<T_{mean}-STD$

T(x,y) is the specific LST values at the local (x,y), $T_{mean}$ and STD are mean value and STD of the LST patterns, respectively.

**Table 4:** Detailed information of impervious surfaces (IS) in the study area from 2002 to 2017.

Year	IS Area (km^2^)	Sprawl Rate of IS
2002	270.75	-
2005	313.04	1.16
2008	373.51	1.19
2011	466.54	1.25
2014	580.70	1.25
2017	678.18	1.17

A dash means no data.

**Table 5:** Statistics of impervious surface sprawl in eight sub-regions from 2002 to 2017.

Sub-Regions	2002–2005	2005–2008	2008–2011	2011–2014	2014–2017	Overall
N	1.08	1.18	1.28	1.20	1.25	2.45
NE	1.22	1.38	1.48	1.28	1.21	3.83
E	1.10	1.19	1.59	1.81	1.36	5.13
SE	1.17	1.45	3.19	2.27	1.49	18.22
S	1.72	1.42	1.39	1.26	1.17	4.98
SW	1.20	1.19	1.24	1.27	1.16	2.58
W	1.05	1.05	1.06	1.04	1.03	1.26
NW	1.21	1.22	1.23	1.27	1.16	2.68

The overall sprawl rate is calculated through dividing the IS area in 2017 by the IS area in 2002.

**Table 6:** Accuracy assessments of extracted LST patterns from 2002 to 2017.

Year	STD	Bias (°C)	CC
2002	0.27	0.33	0.99
2005	0.21	0.29	0.98
2008	0.12	−0.26	0.99
2011	0.23	0.15	0.97
2014	0.30	0.41	0.96
2017	0.20	0.48	0.99

**Table 7:** The sprawl rate of hot thermal landscape (HTL) and urban heat island ratio index (URI) in Wuhan in 2002–2017.

Year	The Area of HTL (km^2^)	Sprawl Tableate of HTL	URI
2002	276.09	-	0.33
2005	298.88	1.08	0.34
2008	369.11	1.24	0.37
2011	433.30	1.17	0.40
2014	460.07	1.06	0.51
2017	531.91	1.15	0.55

A dash means no data.

**Table 8:** Statistics of hot thermal landscape sprawl rate in eight sub-regions from 2002 to 2017.

Sub-Regions	2002–2005	2005–2008	2008–2011	2011–2014	2014–2017	Overall
N	0.63	1.23	1.06	0.91	1.31	0.99
NE	1.07	1.06	2.86	1.19	1.16	4.48
E	-	-	-	-	233.65	233.65
SE	-	-	-	1.85	1.55	2.87
S	1.43	2.21	1.27	1.02	1.01	4.12
SW	1.22	1.15	1.05	1.24	1.10	2.03
W	1.11	1.02	1.02	1.01	1.05	1.22
NW	0.92	1.42	1.31	0.90	1.13	1.73

A dash means that the sprawl rate cannot be calculated by dividing zero. The overall sprawl rates of east sub-region and southeast sub-region are calculated by dividing area in 2017 using area in 2014 and 2011, respectively.

**Table 9:** The statistics of the coupling of LSTWCs and ISFWCs at the city scale.

Year	Distance between LSTWC and ISFWC (m)	Angle Cosine (cos α) between LSTWC Trajectories and ISFWC Trajectories (°)
2002	1944.49	-
2005	2632.78	0.925
2008	2737.43	0.891
2011	3369.70	0.768
2014	5047.76	0.254
2017	3948.09	0.709

A dash means no data.

**Table 10:** The impervious surfaces contribution indexes (ISCIs) in the eight sub-region.

Sub-Region	2002	2005	2008	2011	2014	2017
N	34.52	62.49	58.66	66.61	72.00	105.40
NE	9.71	19.64	22.95	35.71	34.13	66.54
E	2.93	4.20	1.68	10.26	15.83	44.05
SE	0.48	1.09	6.03	20.23	17.43	45.77
S	24.40	55.24	49.27	60.10	77.71	89.63
SW	21.96	59.86	55.93	64.41	112.58	82.12
W	56.73	83.68	65.17	70.63	57.27	55.42
NW	35.45	65.80	59.25	66.98	60.12	74.96

**Table 11:** The variations of ISCIs in the eight sub-region.

Sub-Region	2002–2005	2005–2008	2008–2011	2011–2014	2014–2017	Overall
N	27.97	−3.83	7.94	5.40	33.40	5.42
NE	9.93	3.31	12.76	−1.58	32.41	22.48
E	1.27	−2.52	8.59	5.57	28.22	26.95
SE	0.60	4.94	14.20	−2.80	28.34	27.74
S	30.84	−5.97	10.83	17.61	11.91	−18.93
SW	37.90	−3.93	8.48	48.17	−30.46	−68.36
W	26.95	−18.51	5.46	−13.36	−1.85	−28.80
NW	30.35	−6.55	7.73	−6.86	14.84	−15.51

The overall variations of ISCIs are represented the differences of ISCIs of 2017 and ISCIs of 2002.
